# “Early, rapid, aggressive”: when strategic interactions between governments, opposition, and lobbies can hinder effective responses to epidemics

**DOI:** 10.3389/fepid.2025.1593883

**Published:** 2025-06-18

**Authors:** Alessio Carrozzo Magli, Chris T. Bauch, Alberto d'Onofrio, Piero Manfredi

**Affiliations:** ^1^ Dipartimento di Ricerca Traslazionale e Delle Nuove Tecnologie in Medicina e Chirurgia, Università di Pisa, Pisa, Italy; ^2^ Dipartimento di Economia, Università di Bologna, Bologna, Italy; ^3^ Department of Applied Mathematics, University of Waterloo, Waterloo, ON, Canada; ^4^ Department of Mathematics, Informatics and Geosciences, University of Trieste, Trieste, Italy; ^5^ Dipartimento di Economia & Management, Università di Pisa, Pisa, Italy

**Keywords:** effective responses to epidemics, social distancing, political consensus vs. health protection, political responsibility, game theory, transmission models, preparedness

## Abstract

**Background:**

Two critical factors in the success of the response to a threatening epidemic outbreak are the degree of responsibility of the main political actors involved in the response and the population compliance to the proposed measures. The Behavioural epidemiology literature has focused on the latter factor but largely disregarded the former. The multiple failures in COVID-19 control and the lack of consensus that still surround the main response options (i.e., the elimination-suppression-mitigation trichotomy) highlight the importance of considering the political layer in preparedness activities.

**Methods:**

We integrate a simple transmission model into a game-theoretic framework for the interaction between the main political actors involved in the response, namely a government, its opposition and lobbies. The aim is to provide a conceptual framework allowing one to identify the political factors promoting a timely and effective response.

**Results:**

Low degrees of responsibility (i.e., prioritizing consensus over health protection) of political agents can delay or de-potentiate the response until when epidemic growth eventually overtakes the agents' payoffs, thereby forcing them to switch towards the higher degree of responsibility needed to promote an adequate response. When both the government and the opposition are only “partly” responsible, a stall in the response decision-making process likely arises, further delaying the response. Policy and epidemiological parameters amplifying the response delay are ranked by a sensitivity analysis.

**Conclusions:**

Promoting a high degree of responsibility of political actors and lobbies during emergency situations should be a key target of preparedness. Therefore, future pandemic plans should also include, beyond technical indications, ethical statements “guiding” political entities to cooperation.

## 1 Introduction

Successfully responding to major emergencies, such as a threatening outbreak of a communicable infection, is a critical matter of coordination and responsibility among the different actors involved. Based on the COVID-19 pandemic experience, The Lancet Commission on future preparedness has defined the response to COVID-19 as a global failure at multiple scales where “too many governments have failed to adhere to basic norms of institutional rationality and transparency, too many people have disrespected basic public health precautions, and the world's major powers have failed to coordinate” (1).

In the last two decades, the area of epidemiological modelling has seen the fast development of the behavioural epidemiology (BE) of infectious diseases (2–5). The BE primarily focusses on the role that individuals' risk perceptions and their uncoordinated behaviours can play for the success of public health intervention programmes. This often underlies the tacit hypothesis that, given a well-designed policy (e.g., a vaccination program) conducted by “responsible” policymakers pursuing citizens' health and welfare, the chief threat to citizens' health is represented by their policy resistant behaviour (6). By contrast, the Lancet Commission work has highlighted that, given a certain level of population compliance, the primary factor for a successful response becomes the degree of responsibility and coordination of the main political actors involved (1), a factor typically disregarded in the BE literature.

This work focuses on the role of social distance responses during the early phase of a threatening epidemic, especially when other measures are missing or failing. As repeatedly highlighted (7, 8), this is the crucial epoch where “early, rapid, and aggressive” (ERA) action is critical for the entire response management, at least until the arrival of vaccines. Nonetheless, with the few exceptions of China and other countries which faced the 2003 SARS emergency and that pursued COVID-19 elimination (9–10), such requirements were rarely met during the first COVID-19 wave and many governments around the world were slow to acknowledge the risk and act with urgency (1, 11). This has been especially true, after China, for countries first visited by the COVID-19 pandemic, where heterogeneous, often inadequate, interventions were implemented even after evidence of fully sustained local transmission. This was first the case of Northern Italy, especially in Lombardy region and in its Bergamo province (12), but also of other countries including France, the UK, and the US (11). In the case of Bergamo, the diagnostic systems, hospitals, and ICUs were dramatically overwhelmed, but the extent of the tragedy, with people dying due to COVID without being diagnosed, was initially documented only by TV news showing military trucks carrying away the dead because even cemeteries were overwhelmed. This highly symbolic image of the crisis resonated deeply within the population. The true extent of the epidemic attack was revealed months later, when the excess mortality in Bergamo during March 2020 compared with previous years, was assessed at +600%, with much higher figures recorded in specific municipalities (13).

A critical lesson from this dramatic story is that the ERA principle was not applied and, when the national lockdown was decreed, it was too late to prevent healthcare capacity from being overwhelmed due to COVID-19 characteristics. This occurred despite the evidence from China available from the WHO, the timely alerts from epidemiologists (8) and the availability of strong local public health and economic resources in one of the richest provinces of Italy.

Surprisingly, other countries also intervened tardily despite the tragic story of Bergamo that was unfolding before the world's eyes (14). A main question is therefore why, in many situations, was the ERA principle not applied. Relatedly, little seems to have been done to investigate the potentially harmful role played—at the highest political level—by the strategic interactions between the main actors involved in the response. Evidence from the US states suggests that the political affiliation of the state's governor and external pressures had a larger effect on the hazard of declaring a lockdown than health variables (15). An exemplar is the case of Sweden, where a “mild” policy was chosen, mostly relying on the citizens' social responsibility (16). In the UK, the initial governmental position was to encourage the “do-nothing/herd-immunity” solution (17, 11). Similar delays were pinpointed for France (18–20). All these situations ended when epidemic growth proved so fast that the lockdown declaration was unavoidable and, seemingly, approved by most stakeholders, although at the price of substantially deteriorated control conditions (11).

With regard to the specific case of Bergamo, the news on political events highlighted the pressures enacted by economic lobbies supporting the decision by the Lombardy local government (not opposed by the national government), not to declare hotspots in the area (21), despite clear evidence that a more serious epidemic was ongoing there compared with other areas of Lombardy that were declared hotspots long before. This is still the object of a judiciary inquiry for massacre (21). In addition, ambiguities continued to characterize the statements by opposition parties in Italy (22–25).

Based on previous discussions, we aim to provide a conceptual investigation of the possible roles played by the strategic interactions between the main actors involved in a pandemic response, in determining the timeliness and intensity of measures and subsequent events. More specifically, we combine a game-theoretic framework with three players, namely a government, its opposition, and economic lobbies, with a transmission model, to analyze the implications of the “political behaviour” during a response. In particular, we focus on the mutual ranking that political players attribute to the commitment to protect population health against electoral consensus, what we term their degree of “responsibility,” in weakening or strengthening the application of the ERA principle. Although the work is motivated by the context of the Italian political debate on COVID-19 during the “pre-lockdown” period, it also aims to supply insight for future preparedness as highlighted by the Lancet Commission (1) as well as to provide incitation to the broadening of the borders of BE.

## 2 Methods

We consider the response to a major epidemic threat and the related political game played by an incumbent government with its political opposition and economic lobbies. To keep the exposition as simple as possible, we will make a number of simplifying hypotheses about agents' strategies and their degrees of responsibility. Also, we do not explicitly consider citizens, i.e., voters, in view of their limited power in emergencies, but their preferences are implicitly accounted for in players' decisions, who fear consensus losses at subsequent electoral rounds. However, interested readers can refer to the Supplementary Material (26) where we add several details and derive the model presented here as a stylized representation of a rigorous framework of electoral competition in a democratic system. In this framework, two parties (a “government” and an “opposition”) compete for the consensus of voters while economic lobbies enact their power to move votes.

We distinguish at least two phases in the response period: a “pre-intervention” phase, lasting between the rising epidemic alert and the first evidence of sustained transmission, and a “response” phase, during which interventions are enacted. For example, during the COVID-19 first wave in Italy, the pre-intervention phase ended when the first clusters were discovered in Lombardy and Veneto (20 February 2020), while the national lockdown was implemented between 5 and 22 March (12).

### 2.1 The game-theoretic framework: general hypotheses on political “responsibility”

We consider a game with perfect and complete information, i.e., each player knows the strategies and related payoffs of all other players. The game consists of a sequence of simultaneous one-shot Markov game (27), where agents decide their strategy step by step depending on the current epidemic status. Economic lobbies, instead, simply pursue their interest.

As for the agents' strategies, we assume that the government has the key role in setting up the response to the outbreak (by definition neither the opposition nor the lobby can do this). The government can choose between implementing either a more aggressive policy (strategy “H”) or a milder one (“M”). For simplicity, in our illustrations, we identify policy H as suppression or aggressive containment (8, 10, 28), i.e., one rapidly bringing transmission below threshold by communicating to the population the benefit of such measures with the resulting anticipated economic and social costs. Pairwise, policy M could be crudely identified with mitigation, which typically aims at reducing transmission to slow down the epidemic but not necessarily below threshold and mitigating its impact while imposing less severe socio-economic restrictions (8). Extension to other cases, e.g., any “wait and see” policy (29), is straightforward.

With regard to the opposition, although it does not have roles in the activation of the response, can either choose to encourage (strategy “E”) and support policy H or can oppose it (strategy “N”), by publicly criticizing it and supporting M instead. In the latter case, the opposition can additionally worsen the effectiveness of policy H adopted by the government by encouraging a decline in population adherence to H. With regard to the lobby, we assume that it generally prefers policy M because it fears the damage brought to economic activity by severe closures unless these prove to be unavoidable. We assume that, as the opposition, the lobby can choose whether to encourage policy H (strategy E) or to criticize it (strategy N).

In addition, we assume that the choice of strategies by political actors is mediated by their degree of responsibility towards citizens. We consider three levels of “responsibility”. A government is said to be “responsible,” if it decides to implement policy H regardless of the choice of the opposition and the lobby to encourage it or not. In the language of game theory, a government is responsible if playing H is its dominant strategy. A government is “partly” responsible if it plays H only when supported by the opposition. In other words, its best reaction is H when the opposition supports it (i.e., it plays E), while it is M if the opposition plays N. Formally, neither H nor M is a dominant strategy in this case. Finally, a government is “irresponsible” if playing M is its dominant strategy. The opposition (and the lobby) can be either (i) responsible, if playing E is its dominant strategy, i.e., it will always support the adoption of the aggressive policy; (ii) partly responsible, if its best reaction to H is N and its best reaction to M is E; (iii) irresponsible, if playing N is its dominant strategy.

Plainly, we are considering a scenario where a responsible agent is one always preferring policy H, thereby prioritizing outbreak control over consensus. Likewise, an irresponsible agent will always seek the support of the lobby, thereby sustaining policy M irrespective of the behaviour of its political rival. Instead, partly responsible agents take political considerations (i.e., gaining consensus over the rival) as a priority, although they would prefer policy H over M. Specifically, while preferring policy H, the priority of a partly responsible government is to avoid losing consensus and therefore to obtain political support from the opposition. Similarly, a partly responsible opposition prefers to criticize the government irrespective of the policy actually implemented, although it prefers that the government implements policy H. In other words, partly responsible agents are not (entirely) controlled by the lobby but prioritize not to concede any political advantage to the rival. Further details on the concept of responsibility of political actors based on a general framework of election competition are reported in the Supplementary Materials (26) and the underlying theory (30).

The agents' strategic interactions are analyzed first in the pre-intervention phase where the net benefits of the adopted policies—payoffs in game theory language—are fully exogenous, i.e., independent of infection incidence. In this phase, the agents' *a priori* opinions about epidemic risks combine with the general political debate of ordinary periods. The analysis of the pre-intervention phase will identify the role played by the agents' degree of responsibility on the possibility that the system will implement or not policy H as soon as evidence of sustained transmission becomes available. Next, we consider the response phase, during which players will include the perceived (direct and indirect) costs of the epidemic into their payoffs.

### 2.2 General payoff matrices for the political game in the pre-intervention phase

Games are typically represented through their payoff matrix (27) which reports the *net utilities,* or “payoffs” in game theory language, resulting for any given combination of strategies chosen by players. Payoffs will have the general form $P_{a}^{\;period}(i,j,h)$ where *a* denotes the agent (*a* = government, opposition, lobby), period refers either to the pre-intervention (pre for brevity) or to the response phase, respectively, and indices i,j,h refer to the agents' possible strategies (i=H,M;j=E,N;h=E,N). The resulting 2 × 2 × 2 payoff array can be simplified (details in Supplementary Material, Part 1) under the realistic hypothesis that N is the lobby's dominant strategy. This means that the lobby's best reaction is always supporting strategy M, regardless of the strategy adopted by the opposition.

This said, from now on we will, for sake of notational simplicity, align the strategy sets across all actors by using the simple argument that if the opposition is encouraging policy H (M) adopted by the government, it means that H (M) is also the preferred policy of the opposition. Pairwise, M will always be the lobby's dominant strategy. Therefore, the final payoff matrix of the pre-intervention phase, will have the (2 × 2) symbolic form in Table 1.

**Table 1:** The (2 × 2) representation of agents' payoff matrix during the pre-intervention phase under the hypothesis that M is the lobby's dominant strategy, i.e., the lobby always plays M.

Government	Opposition (with lobby always playing M)	
	H	M
H	$P_{gov}^{\;pre}(H,H,M)$	$P_{gov}^{\;pre}(H,M,M)$
	$P_{opp}^{\;pre}(H,H,M)$	$P_{opp}^{\;pre}(H,M,M)$
	$P_{lobby}^{\;pre}(H,H,M)$	$P_{lobby}^{\;pre}(H,M,M)$
M	$P_{gov}^{\;pre}(M,H,M),$	$P_{gov}^{\;pre}(M,M,M)$
	$P_{opp}^{\;pre}(M,H,M),$	$P_{opp}^{\;pre}(M,M,M)$
	$P_{lobby}^{\;pre}(M,H,M)$	$P_{lobby}^{\;pre}(M,M,M)$

Payoffs in each cell are ordered as follows: Government (first row), opposition (second row), and lobby (third row).

Concretely filling the payoff matrix depends on the degrees of responsibility of the three political actors and will be done at a later stage. Briefly, each actor will rank the four possible combinations of strategies in Table 1, namely, (H,H,M),(H,M,M),(M,H,M),(M,M,M), in terms of the benefit they receive from that particular combination. We denote by A≥B≥C≥D the corresponding payoffs ranked from the first best (A) to the worst case (D).

### 2.3 Epidemiological model

To keep a high level of generality, we do not stick to any specific infection and rather consider a simple model of infection and severe disease. As our focus is on early response to a threatening outbreak, we linearize by assuming that the depletion of the susceptible compartment is negligible. This means that (i) the susceptible fraction *S* does not significantly depart from 100%, and can therefore be disregarded; (ii) the number Y(t) of infective individuals at any time *t* obeys the linear ordinary differential equation $Y^{\prime }(t)=\gamma (R(t)-1)Y(t)$, where γ denotes the recovery rate and R(t) is the reproduction number of the infection at time *t*. In particular, R(t) is a piecewise constant function that takes on value $R_{0}$, i.e., the infection basic reproduction number, during the pre-intervention phase of free epidemic growth lasting from time zero to time $t=t_{0}$. Instead, during the response phase, R(t) can either take on value $R_{H}$ or $R_{M}$ depending on which control policy (H and M) the government chooses to implement. The quantity $R_{H}$ $\left(R_{M}\right)$ is the *control reproduction number of policy H (M),* representing the number of secondary cases caused by an infective individual in a wholly susceptible population under that policy. We assume that the adopted policy, once declared at time $t_{0}$ is implemented by immediately bringing transmission to the desired level $R_{i}\;(i=H,M)$. The proposed formulation, which implies epochs of exponential growth/decline of Y(t) while keeping the susceptible fraction nearby 100%, was termed the *low-attack rate* (LAR) hypothesis in (31, 32), where it proved useful to describe the first two years of the COVID-19 epidemic in Italy. The LAR hypothesis reflects the idea that in the absence of vaccines, threatening outbreaks potentially overwhelming public health capacities, cannot be left free to generate large attack rates.

In addition, we assume that a certain fraction $\rho =\rho_{1}\gamma \;(0<\rho_{1}<1)\;$ of infection cases eventually end into severe disease (requiring a costly treatment) with an appropriate delay distribution of disease onset. Formally, letting the dynamic variable Z(t) represent the cumulative incidence of severe cases at time *t*, the model reads

(1) $$Y^{\prime }(t)=\gamma (R(t)-1)Y(t),$$

(2) $$Z^{\prime }(t)=\rho \;\cdot \int_{0}^{\infty }Y(t-\tau )K(\tau )d\tau ,$$

where *K* is a probability density function representing the “disease generation” distribution given infection. Letting

(3) $$U(t)=\int_{0}^{\infty }Y(t-\tau )K(\tau )d\tau ,$$

and assuming that *K* obeys an exponential distribution with scale $T_{A}=1/A$, where *A* is the corresponding decay rate, the model reduces to the following system of three ordinary differential equations:

(4) $$Y^{\prime }(t)=\gamma (R(t)-1)Y(t),$$

(5) $$U^{\prime }(t)=A(Y(t)-U(t)),$$

(6) $$Z^{\prime }(t)=\rho \;\cdot U(t).$$

Note that the scale parameter $T_{A}=1/A$ represents the average delay between infection and onset of severe disease.

### 2.4 General payoff matrices during the response phase

At the onset of the response phase, which starts after evidence of sustained transmission (at time $t=t_{0}$), the government will intervene by adopting either policy *H* or policy *M*. Since this moment, payoffs of all actors will account for perceived epidemic costs. The latter are taken as the sum (having negative sign) of the direct cost of the epidemic, arising from cases of severe disease, and of its indirect cost due to the impacts of control policies (H or M) on economic activity and the society as a whole.

Assuming that the government has adopted policy *i*, we take as a baseline the case where the corresponding direct costs $\left[DC_{i}(t)\right]$ and indirect costs $\left[IC_{i}(t)\right]$ are not affected by the strategy adopted by the opposition. Given the epidemiological model (Section 2.3), direct costs of policy *i* are assumed to reflect the cumulative cost due to cases of serious disease [Z(t)] (Section 2.3) under that policy though a cost parameter $q_{DC}$:

(7) $$DC_{i}(t)=q_{DC}\int_{t_{0}}^{t}Z_{i}(u)du.$$

Indirect costs can be taken, in the simplest case, as proportional (through cost parameter $q_{IC}$) to the difference between epidemic reproduction without intervention, reflecting “normal” socio-economic activity, and reproduction under policy *i*, reflecting “altered” activity, that is

(8) $$IC_{i}(t)=q_{IC}\int_{t_{0}}^{t}(R_{0}-\;R_{i})dt=q_{IC}(R_{0}-\;R_{i})(t-t_{0}).$$

In addition, the three players will weigh the two cost items differently, with $0<\alpha_{G}<1,\;0<\alpha_{O}<1,\;0<\alpha_{L}<1$, representing the agents' specific preference for direct costs. Our general hypotheses imply that $\alpha_{G}>\alpha_{L}$, $\alpha_{O}>\alpha_{L}$ (i.e., the lobby is biased towards indirect costs). Instead, $\alpha_{G}$ and $\alpha_{L}$ will obey the same ranking as the corresponding degree of responsibility, i.e., if the government is responsible and the opposition partly, then $\alpha_{G}>\alpha_{O}$. The resulting payoff matrix of the political game in the response phase is reported in Table 2. This describes a baseline situation where the government, while implementing the chosen policy *i*, has full control over the ensuing epidemic course regardless of the strategy adopted by the opposition. Arguably, an opposition choosing policy M and therefore criticizing a government adopting policy H, might also affect costs. For example, if this criticism is used to attack the governmental policy H over public media by emphasizing the distress caused to the population by the government's measures, the individuals' compliance to governmental actions could decrease raising direct costs and also indirect costs might be affected. Although this is a possible realistic complication of our framework, we will not consider it for the sake of simplicity and will stick to the baseline case.

**Table 2:** General structure of the payoff matrix prevailing during the response phase under the baseline assumption that the government has full control of the epidemic course regardless of the strategy (encouraging or criticizing) followed by the opposition.

Government	Opposition (with Lobby always playing M)	
	H	M
H	$P_{gov}^{\;pre}(H,H,M)-\alpha_{G}DC_{H}(t)-(1-\alpha_{G})IC_{H}(t),$	$P_{gov}^{\;pre}(H,M,M)-\alpha_{G}DC_{H}(t)-(1-\alpha_{G})IC_{H}(t),$
	$P_{opp}^{\;pre}(H,H,M)-\alpha_{O}DC_{H}(t)-(1-\alpha_{O})IC_{H}(t),$	$P_{opp}^{\;pre}(H,M,M)-\alpha_{O}DC_{H}(t)-(1-\alpha_{O})IC_{H}(t),$
	$P_{lobby}^{\;pre}(H,H,M)-\alpha_{L}DC_{H}(t)-(1-\alpha_{L})IC_{H}(t)$	$P_{lobby}^{\;pre}(H,M,M)-\alpha_{L}DC_{H}(t)\;-(1-\alpha_{L})IC_{H}(t)$
M	$P_{gov}^{\;pre}(M,H,M)-\alpha_{G}DC_{M}(t)-(1-\alpha_{G})IC_{M}(t),$	$P_{gov}^{\;pre}(M,M,M)-\alpha_{G}DC_{M}(t)-(1-\alpha_{G})IC_{M}(t),$
	$P_{opp}^{\;pre}(M,H,M)-\alpha_{O}DC_{M}(t)-(1-\alpha_{O})IC_{M}(t),$	$P_{opp}^{\;pre}(M,M,M)-\alpha_{O}DC_{M}(t)-(1-\alpha_{O})IC_{M}(t)$
	$P_{lobby}^{\;pre}(M,H,M)-\alpha_{L}DC_{M}(t)-(1-\alpha_{L})IC_{M}(t)$	$P_{lobby}^{\;pre}(M,M,M)-\alpha_{L}DC_{M}(t)-(1-\alpha_{L})IC_{M}(t)$

Payoffs in each cell are ordered as follows: government (first row), opposition (second row), and lobby (third row).

### 2.5 Analysis of the political game of outbreak response

Let us focus first on the pre-intervention game (Table 1). Given the three levels of responsibility of the three political actors, the general form of Table 1 can be specified into 3 × 3 × 3 = 27 distinct political games. However, still due to the hypothesized role of the lobby, these 27 subcases eventually collapse into nine games only (proof in Supplementary Material, Part 1). These nine games are described by the pairs (1) (Resp,Resp), i.e., a responsible government playing against a responsible opposition; (2) (Resp,Partly), i.e., a responsible government against a partly responsible opposition; (3) (Resp,Irr), i.e., a responsible government against an irresponsible opposition, etc.

These nine distinct games differ in their payoff matrices (Tables 1,2). Given the role of the lobby, this depends on the way the government and the opposition rank their ordinal preferences over the four possible outcomes of their 2 × 2 game: (H,H), (H,M), (M,H), (M,M). Once the payoff matrix is assigned, the key concept to the solution of the game is that of Nash equilibrium (33, 34). A Nash equilibrium emerges when no player has the incentive for an *ex post* deviation from the selected strategy (after observing the strategies chosen by all other agents) because whatever change would worsen their reward. Remarkably, not all game-theoretic models have a unique Nash equilibrium. There are situations where, e.g., no Nash equilibrium exists in *pure strategy* (i.e., at least one player might randomize over two or more alternative but indifferent strategies), or where multiple equilibria coexist. A relevant case for our analyses is the so-called *discoordination game* (33) where both political actors cyclically have an *ex post* incentive to change strategy after observing the choice of the rival, resulting in a Nash equilibrium in which all outcomes might occur with positive (but not 100%) probability. Notably, a discoordination game reflects a situation where the decision-making process has stalled. Basic game theory tackles this problem by randomization, i.e., every new time step players choose their strategy by “flipping a coin” whose success probability will reflect the game's payoff. As we will see later on, the stall will cause a delay in the activation of the response. However, epidemic growth will modify the success probability *p* of the opposition over time. Therefore, when this probability reaches zero, the government will not anymore be indifferent between H and M and the pure strategy Nash equilibrium of the game re-emerges.

Seeking solutions to the full political game requires, in principle, to analyze all the aforementioned nine different games arising for the pre-intervention phase. However, it can be shown (see Supplementary Material, Part 1) that there are three pivotal games that allow one to easily derive the solutions to all cases. These “key” games are those played by a partly responsible government against the possible degrees of responsibility (responsible, partly responsible, irresponsible) of the opposition. These pivotal cases will be carefully discussed in the Results section.

### 2.6 The response phase: model parameters and sensitivity analysis

With regard to the response phase (Table 2), we investigate, by one-parameter and multi-parameter sensitivity analyses, the dependence of the main model outputs namely, the delay of enaction of policy H and the legacy of policy M, on political and cost parameters while holding the epidemiological parameters constant. Cost parameters include the *a priori* payoffs of the pre-intervention phase (i.e., quantities A, B, C, D), the weights attributed by political actors to direct costs ($\alpha_{G},\;\alpha_{O}$, $\alpha_{L}$), the unit cost of a case of severe disease $\left(q_{DC}\right)$ and the socio-economic cost of a day of restrictions $\left(q_{IC}\right)$. The *a priori* payoffs, which embed the perceived costs of an incoming epidemic but also ordinary political costs, were assumed to be crudely comparable to the estimated financial costs needed for electing and keeping on a government team (35). The baseline for indirect costs $\left(q_{IC}\right)$ was taken as the daily Italian GDP (35). The cost of a case of severe disease $\left(q_{DC}\right)$ was taken of the magnitude of the current discounted costs of a lifetime disability (preventing income earnings and causing caring costs thereafter). In view of the highly stylized nature of the adopted model of infection transmission and disease, epidemiological parameters were set to crudely depict an epidemic spreading rapidly in the absence of control. In particular, we take a basic reproduction number $R_{0}$ of 2.5 as a compromise between typical estimates from pandemic flu [as, e.g., $R_{0}=1.8$ reported in (36)] and those reported for Italian regions during the first COVID-19 wave (37, 38). The duration of infection $\gamma^{-1}$ was set to 5 days. Combined with the value of $R_{0}$, this implies a short epidemic doubling time $h_{D}$ (about 2.3 days). The average delay $T_{A}$ between infection and severe disease was set to a baseline of 10 days and the risk of serious disease given infection to ρ=0.01. The epidemic is initialized from Y(0) = 20 infective seeds at the start of the pre-intervention phase (t=0). Given the uncertainty in cost parameters, we used a sensitivity analysis to cope for uncertainty giving a qualitative feeling of the main influences. Model parameters are summarized in Table 3. Parameters specifically used in the sensitivity analyses are (Table 3, column 4): (a) the epidemic reproduction $\left(R_{M}\right)$ under policy M, (b) the delay $\left(T_{A}\right)$ between infection and severe disease, (c) the duration $D_{\;pre}=t_{0}$ of the pre-intervention phase, (d) the scale (Q) of indirect vs. direct unit costs, (e) the scale of pre-response payoffs, and (f) the government weight for direct costs.

**Table 3:** Full list of model parameters, their baseline values, and ranges of free parameters for the sensitivity analysis.

Parameter	Description	Baseline value	Free simulation parameters: range for sensitivity analysis
$R_{0}$	Basic reproduction number of infection	2.5	
$R_{M}\;$	Control reproduction number of policy M	1.2	[1.05, 1.9]
$R_{H}$	Control reproduction number of policy H	0.7	
$\gamma^{-1}$	Duration of infection	5 days	
$T_{A}$	Delay of onset of serious disease given infection	10 days	[2.5,25]
ρ	Rate of onset of serious disease given infection	0.01 day^−1^	
$D_{\;pre}$	Duration of pre-intervention phase	15 days	[15,35]
$w_{Gov}$	Government weight for direct cost	0.5	[0,1]
$w_{Opp}$	Opposition weight for direct cost	0.5	
$q_{DC}$	(Direct) Cost of a case of serious disease in rescaled unity	1	
$q_{IC}$	(Indirect) Cost of one day of altered socio-economic activity	$Q\cdot q_{DC}$	
Q	Cost of one day of altered social activity relative to a case of serious disease	100	[50,500]
[A,B,C,D]	Payoffs of the four policy options during the pre-intervention phase	P⋅[4,3,2,1]	
P	Parameter scaling the level of pre-intervention payoffs	1,000	[500:5,000]
q,p	Mixed-strategy probabilities in the discoordination game	[0,1]	

## 3 Results

We first provide general results of the political game in the pre-intervention phase, offering detailed intuitions of its conceptual outcomes for the three pivotal cases. Then, we analyze the response phase by integrating epidemic trends into the political game.

### 3.1 The pre-intervention phase

The possibility that policy H is immediately enacted after first evidence of sustained transmission (i.e., a true ERA policy) depends on the degrees of responsibility of the government and the opposition:

• Policy H will be immediately adopted when the government is responsible (regardless of other agents) and also when it is only partly responsible, provided it faces a responsible opposition.
• Policy H will not be adopted (M is adopted instead) if the government is irresponsible or if it is partly responsible but the opposition is irresponsible. This will delay the enaction of H and the extent of the delay will depend on the trend of the government payoff during the response phase.
• A stalled decision-making arises if both the government and the opposition are partly responsible.

A proof of the previous statement is reported in Supplementary Material, Part 1.

In what follows, we discuss in depth the three “pivotal” games mentioned in Section 2.6, namely those played by a partly responsible government against the possible degrees of responsibility of the opposition.

The analysis requires to first specify the corresponding payoffs in the general payoff matrix of Table 1. First, we assign the payoffs of a partly responsible government, i.e., a government that prioritizes the pursuit of consensus over epidemic control. The best outcome (payoff A) is implementing policy H with the support of the opposition (which also prioritizes H), because this yields the best health outcome without consensus losses. The second best (payoff B) is implementing policy M without being criticized by the opposition and lobby (also playing M), i.e., safeguarding consensus despite poorer outbreak control. The third best (payoff C) is implementing H suffering the critics of the opposition and lobby (playing M). The worst outcome (payoff D) occurs when it implements policy M and is publicly attacked for it (because the opposition plays and “invokes” H), therefore yielding a lower epidemic control while losing consensus. As stated in the methods, it holds, A≥B≥C≥D. The payoffs of the opposition (and lobby) are assigned below while presenting the three pivotal subcases.

#### 3.1.1 First pivotal subcase: a “partly” responsible government against a responsible opposition

Before completing the filling of the payoff matrices, it is important to make the following terminological clarification due to the different roles of the government and the opposition. When it is stated that the government is playing, e.g., strategy M, it also means that it is actually implementing that policy. Instead, when the opposition (and lobby) “plays,” e.g., strategy M, it simply means that they are just publicly claiming that the government should implement that policy or that they would implement it if they could form a new government.

In this case, unlike the government, the opposition prioritizes epidemic control over consensus. Its best outcome (payoff A) is playing policy H, while the second best (payoff B) is playing policy M while criticizing policy H adopted by the government. The third best (payoff C) is to criticize the government for adopting M (note that the second and third best could be switched without affecting the results). The worst outcome is encouraging (M) a government implementing policy M, thereby achieving a lower epidemic control with no political advantage. By matching the payoffs of the opposition with those of the government for each combination of strategies, we obtain the corresponding payoff matrix (Table 4). The configuration (H,H) emerges as the game (unique) Nash equilibrium. Therefore, the government will implement strategy H (despite the lobby's criticism) without delay and the opposition will publicly sustain it, thereby not exploiting the emergency for political consensus. The ERA policy is therefore the outcome of the political game. Having postulated the ERA policy as the best societal outcome, citizens (together with the government and opposition parties representing them) are the “winner” of the game despite the fact that they did not play it, while the lobby is the “loser.”

**Table 4:** The pre-intervention phase.

Government	Opposition (and Lobby)	
	H	M
H	A,A,C	C,B,C
M	D,C,A	B,D,A

Payoff matrix for the strategic interaction between a “partly responsible” government and a “responsible” opposition yielding to the unique Nash equilibrium (H,H). Payoffs are reported in each cell according to the following order: government, opposition, and lobby and obey A≥B≥C≥D.

#### 3.1.2 Second pivotal subcase: a partly responsible government vs. an irresponsible opposition

The first best (payoff A) of an irresponsible opposition will be invoking policy M and criticizing the government for adopting H, because the better epidemic control will be counterbalanced by higher societal costs while simultaneously obtaining the consensus of the lobby. As second best (B), the opposition prefers to criticize the government for implementing policy M, i.e., to invoke policy H (this ordering can be interchanged without affecting the results). Similarly, the third (C) and fourth best (D) occur when the opposition encourages the government for implementing policy H and M, respectively, by playing the same strategy. From the payoff matrix (Table 5), one notes that M is both the dominant strategy of the opposition and the ensuing best reaction of the government. Therefore, the only Nash equilibrium is (M,M), meaning that the ERA policy will not be implemented because the milder policy M is preferred. Here, the true winner is the lobby, which is able to control both the government and the opposition, while the losers are the citizens.

**Table 5:** The pre-intervention phase.

Government	Opposition (and lobby)	
	H	M
H	A,C,C	C,A,C
M	D,D,A	B,B,A

Payoff matrix for the strategic interaction between a “partly responsible” government and an “irresponsible” opposition (and lobby) yielding to the unique Nash equilibrium (M,M). Payoffs are reported in each cell according to the following order: government, opposition, and lobby and obey A≥B≥C≥D.

#### 3.1.3 Third pivotal subcase: both the government and the opposition are partly responsible

The first best of a partly responsible opposition would be to criticize H and to support M instead; the second best would be to criticize M by supporting H (note the ordering of these two alternatives can be switched without altering the content of the game). The third and fourth bests emerge when the opposition supports the same policy of the government, with (H,H) being preferred to (M,M) (Table 6). Notably, this results in a discoordination game where the government tries to coordinate with the opposition while the latter tries to avoid it. In such a game, no pure strategy Nash equilibrium exists and a stall in decision-making (Section 2.5) appears. If the game could be played repeatedly during the pre-intervention phase, a “cyclic” behaviour would emerge where both the government and the opposition randomly change their intentions towards control policies along a random walk because the only Nash equilibrium is in mixed strategy, i.e., both players cannot but “randomize” their behaviour, with the government intending to implement H with probability $q=\frac{B-D}{\left(A+B-C-D\right)}$ and the opposition supporting it also invoking H, with probability $p=\frac{B-C}{\left(A+B-C-D\right)}<q$. Randomization means that the outcome of the single-shot of the game can be any of the four possibilities listed in Table 6, ranging from the “desirable” (H,H) (with probability qp) to the “bad” (M,M) [with probability (1 − q)(1 − p)] (details in the Supplementary Material).

**Table 6:** The pre-intervention phase.

Government	Opposition (and lobby)	
	H	M
H	A,C,C	C,A,C
M	D,B,A	B,D,A

Payoff matrix for the strategic interaction between a “partly responsible” government and a “partly responsible” opposition (and the lobby). Payoffs are reported in each cell according to the following order: government, opposition, and lobby and obey A≥B≥C≥D.

### 3.2 The response phase and delays of implementation of the ERA policy

We now discuss the implications that the political games involving a partly responsible government (Section 3.1) have on the management of the response phase. Since we proved (Subsection 3.2.1) that the ERA policy H was adopted without delay in the case of a partly responsible government and a responsible opposition, we focus on the cases where the opposition is either irresponsible or partly responsible.

#### 3.2.1 A partly responsible government vs. an irresponsible opposition: time at lockdown and M-legacy

The presence of an irresponsible opposition entraps the pre-intervention political game in the (M,M) Nash equilibrium (Section 3.1.2), forcing the government to adopt policy M. However, epidemic growth under M is expected to cause direct cost to eventually overwhelm indirect ones. Therefore, switching to H is eventually expected when the payoff of H will exceed the one of M, i.e., when the incremental payoff of M ($\Delta Payoff_{MH}$, also termed the “payoff gain”) with respect to H becomes negative. By combining the corresponding pre-intervention payoffs (Table 5) with the outbreak costs (Table 2), this occurs for

(9) $$\Delta Payoff_{MH}=B-C-\alpha_{G}(DC_{M}(t)-DC_{H}(t))-(1-\alpha_{G})(IC_{M}(t)-IC_{H}(t))\leq 0.$$

**Figure 1:**
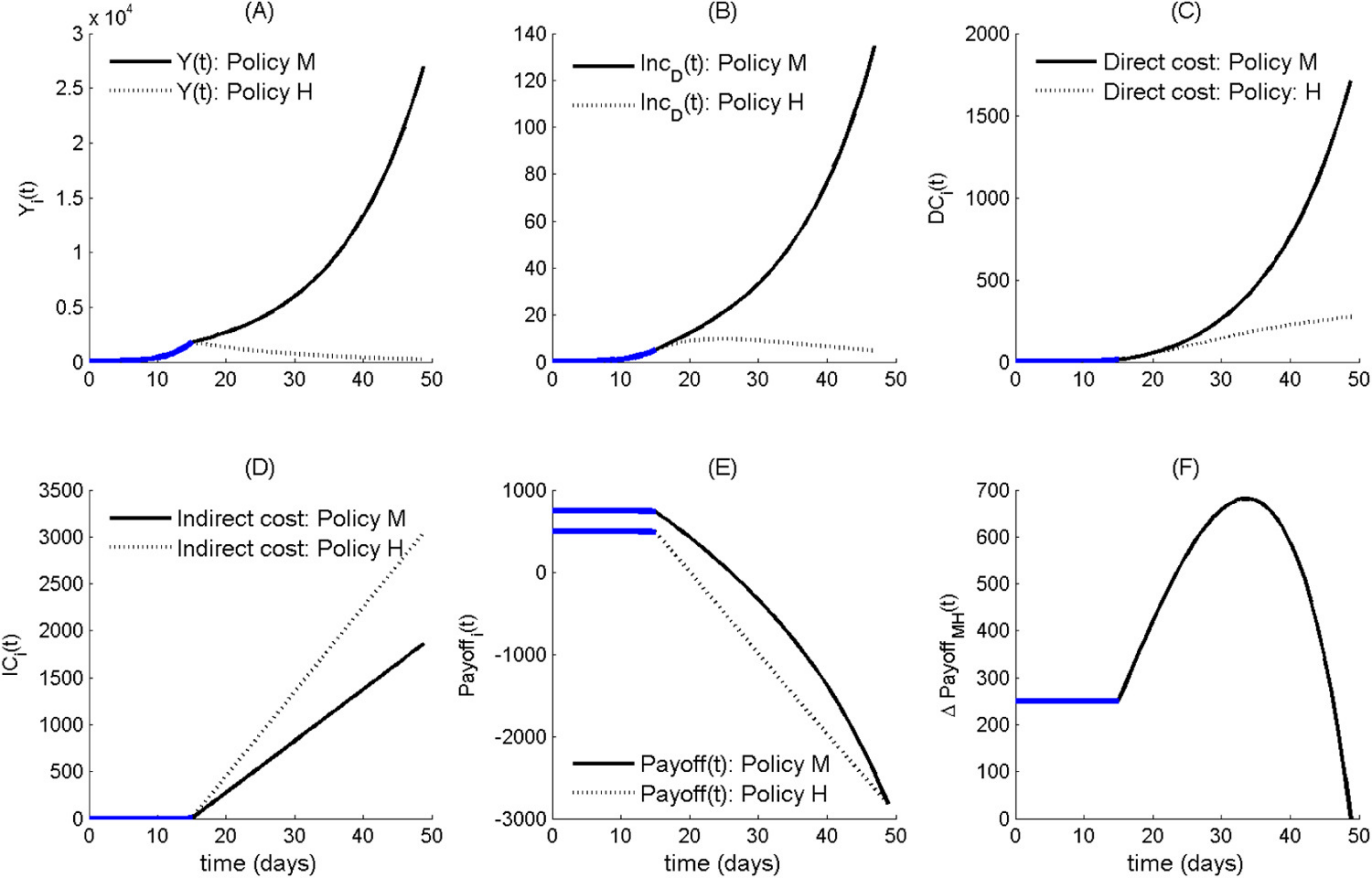
The case of partly responsible government vs. an irresponsible opposition initially entrapping the political game on the M policy and causing a delayed switch to policy H. The different subgraphs report, along the two different policy options (M vs. H), the temporal trends of **(A)** epidemic prevalence Y(t), **(B)** incidence of severe disease cases, **(C)** direct epidemic costs, **(D)** indirect costs, **(E)** government payoffs; and **(F)** $\Delta Payoff_{MH}$. The thick blue traits in each subgraphs denote trends during the pre-intervention phase. All parameter values are set at their baseline level (Table 3).

Given that the government is partly responsible, it holds $0<\alpha_{G}<1$ ($\alpha_{G}=0$ holding only for an irresponsible government, $\alpha_{G}=1$ only for a responsible one), so the switch to H policy is expected to always occur sooner or later (Figure 1).^1^ After the free growth of infective and severe cases during the pre-intervention phase (during which payoffs of both policies are constant), the adoption of policy M (Figures 1A,B) at the start of the response phase yields rapidly diverging direct costs of the two policies (Figure 1C). Given the linear trend of indirect costs (Figure 1D), the gap between payoffs (Figure 1E) eventually annihilates. At the switch time $t_{s}$ when $\Delta Payoff_{MH}$ vanishes (“time-to-lockdown”, TTL) the switch to policy H occurs (Figure 1F). We pinpoint the “reversed U” behaviour of $\Delta Payoff_{MH}$, which initially increases due to the expanding gap of indirect costs (linear) over direct ones. That is, the relationship between costs can have a key role in delaying the transition to policy H. A further potential shortcoming is that political behaviour could attempt to exploit this initial increase of the M payoff to further support policy M among citizens, who are arguably unaware of the underlying political game. Although this further behavioural aspect is not included in the model, during the early COVID-19 epoch in Italy, the argument “no more than a trivial influenza…” was systematically invoked to promote milder actions.

**Figure 2:**
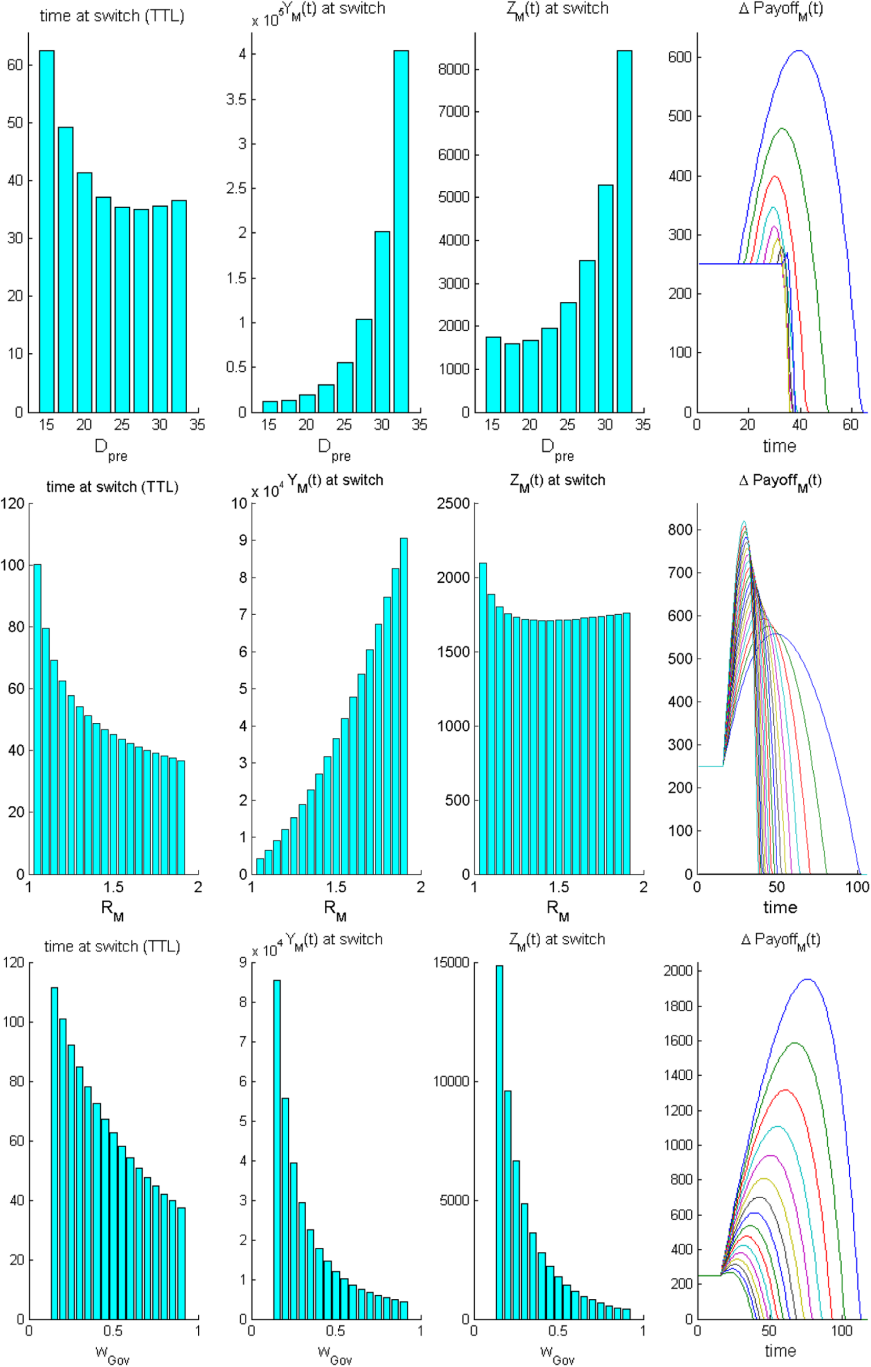
The case of a partly responsible government vs. an irresponsible opposition during the response epoch: one-parameter sensitivity analyses of the time at switch $\left(t_{S}\right)$ from policy M to policy H and related main epidemiological outputs. Top row: sensitivity to the duration of the pre-intervention phase $D_{\;pre}$. Central row: sensitivity to $R_{M}$. Bottom row sensitivity to the governmental preference towards protection of direct costs, $w_{Gov}$. Each row reports from left to right: **(A)** time to switch $\left(t_{S}\right)$, **(B)** epidemic prevalence at $t_{S}$, $Y(t_{S})$, **(C)** cumulative number of severe cases at $t_{S}$, $Z(t_{S})$, and **(D)** temporal trend of the $\Delta Payoff_{MH}$. Other parameter values are set at their baseline level (Table 3). The interpretation of trends of $\Delta Payoff_{MH}$ is provided in the main text.

Figure 2 reports one-parameter sensitivity analyses of the TTL (first column) and of the related “legacy” of policy M to the incoming policy H in terms of both infection prevalence at switch time [$Y(t_{S}),\;$ second column] and of cumulative cases of severe disease [$Z(t_{S})$, third column], with respect to some main policy parameters, namely, (i) the duration $\left(D_{Pre}\right)$ of the pre-response phase of free epidemic growth (upper panels), (ii) the intensity of epidemic reproduction under policy M ($R_{M}$, central panels), and (iii) the magnitude $w_{Gov}$ of the government prioritization of direct costs (bottom panels). The time trends of the $\Delta Payoff_{MH}$ (fourth column) assist the outputs' interpretation.

First, increasing durations $D_{\;pre}$ of the pre-intervention phase (Figure 2, top panels) yield a U-shaped effect on TTL and severe disease. The initial decline in the TTL occurs because increasing $D_{Pre}$ values cause higher levels of the epidemic curve at intervention onset which, in turn, reduce the initial growth phase of the net payoff due to indirect costs. However, large $D_{\;pre}$ values prevent the initial growth of $\Delta Payoff_{MH}$ because direct costs become massive. In this case, any further increase in $D_{\;pre}$ more than counterbalances the opposite effect on costs, thereby increasing the TTL. Correspondingly, also the legacy to H is U-shaped. Indeed, under an early start of the response phase, the initial growth of indirect costs is disproportionate so that the TTL will, other being equal, increase and the resulting (cumulative) number of severe cases will worsen.

Increasing values of the control reproduction number of policy *M* ($R_{M}$, Figure 2, central panels) promote (other things being equal) smaller and smaller levels of the TTL with a dramatic increasing legacy in terms of infection prevalence (this is expected due to the faster epidemic growth). However, this also yields a U-shaped relationship with the cumulative number of serious cases. This is also due to the effects that different $R_{M}$ values have on the $\Delta Payoff_{MH}$ curves. Interestingly, in this case, the number of severe cases is high for large values of $R_{M}$ (as expected), but it is also relatively high when $R_{M}$ is low, because in this case, the slow accumulation of direct costs will cause policy H to be adopted lately, allowing cases of severe disease to cumulate substantially.

Finally, increasing values of the government's preference $w_{Gov}$ towards direct costs (Figure 2, bottom panels) will cause sharp reductions in the initial boom of the net payoff of policy M (bottom right panel). This promotes, other things being equal, a marked decline in the TTL, which, in turn, associates with dramatically decreasing levels of both infection prevalence [Y(t)] and cumulative cases of disease [Z(t)] at $t_{s}$.

**Figure 3:**
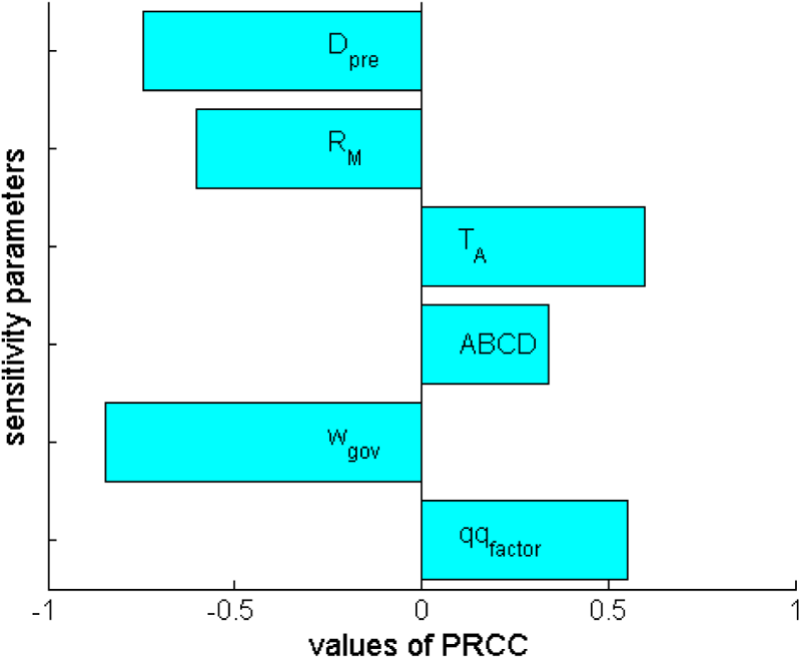
The response epoch for the case of a partly responsible government vs. an irresponsible opposition: sensitivity analysis based on partial rank correlation coefficients (PRCC) of the time at switch from M to H policy with respect to critical policy parameters, namely, (1) the duration of the pre-intervention phase $\left(D_{\;pre}\right)$, (2) the intensity of transmission under policy $M(R_{M})$, (3) the delay of appearance of serious disease with respect to infection $\left(T_{A}\right)$, (4) the scale of pre-intervention payoffs (ABCD), (5) the governmental preference $\left(w_{Gov}\right)$ for direct costs, and (6) the factor of indirect cost relative to direct ones. Parameter ranges are those in Table 3.

The overall sensitivity of the switch time to policy H with respect to the main policy parameters (Figure 3) shows that (i) both the preference attributed by the government to direct costs $\left(w_{Gov}\right)$ and the duration $D_{\;pre}$ of the initial pre-intervention phase are strongly negatively correlated with the TTL (more than 80% and about 75%, respectively); (ii) also the magnitude of $R_{M}$ shows a negative, although less pronounced, correlation with the TTL; (iii) the delay of onset of severe disease after infection, the relative indirect cost and the pre-intervention payoffs positively affect the TTL (the latter in a less pronounced way, as expected).

Once the epidemic trend has forced the enaction of policy H, the question obviously becomes for how long policy H will be sustainable, given its larger indirect costs compared with M. In this simple framework, based on the LAR hypothesis (i.e., the epidemic curve under policy M remains exponentially increasing at the same speed), a new payoff switch restoring policy M would require the inclusion of a non-linear term in the indirect costs, possibly reflecting societal and psychological distress, or “fatigue” (39) caused by enduring closures. In the latter case, all the effects illustrated so far, remain true. For example, a lower weight attributed to direct costs, that caused a delayed switch to policy H, would additionally—during the responses phase—cause a more rapid growth of indirect costs up, calling for an earlier re-switch to policy M. This could occur long before appropriate epidemic control targets are reached.

#### 3.2.2 Both the government and the opposition are partly responsible: persistence of stalling decision-making

The stall in decision-making prevailing in the pre-intervention phase, extends to the response phase because the political game remains dis-coordinative, at least initially. Remarkably, while the decision power lies in the government's hands, it is the opposition degree of responsibility that determines the subsequent evolution of the game. We basically distinguish three possibilities. First, policy H is expected to be adopted and persist over time (i.e., after repeated randomizations) if probability *q* is close to unit (say, greater than 95%). This requires that the opposition, although partly responsible, ranks citizens' health higher than consensus. Formally, the difference A-C shall be low enough [with A=C as the limit case distinguishing between a partly and a (fully) responsible opposition]. Second, policy M is expected to be adopted and persist over time if probability *q* is very small (say, lower than 5%). This requires that the opposition prefers gaining political consensus over preserving citizens' health. Formally, the difference B−D shall be low enough (with B=D as the limit case distinguishing between a partly responsible and an irresponsible opposition). Notably, after policy M has been adopted, this case will work as in the previous subsection.

Last, there are the intermediate situations where q lies in an intermediate probability range. In the latter case, a “schizophrenic” control policy would emerge where the government continuously calls for strengthening (H) or reducing (M) restrictions based on tossing a coin and, pairwise, the opposition continuously switches between encouraging and criticizing the government irrespective of the ongoing epidemic activity. This control policy would eventually come to an end because in the epidemic phase, the costs of the outbreak will modify the mixed-strategy probabilities. Specifically**,** the onset of sustained transmission modifies the probability *p* that the opposition will support policy H such that the government is indifferent between implementing H or not, as follows:

(10) $$p=\frac{B-C-\alpha_{G}DC_{M}(t)-(1-\alpha_{G})IC_{M}(t)+\alpha_{G}DC_{H}(t)+(1-\alpha_{G})IC_{H}(t)}{\left(A+B-C-D\right)}.$$

This means that during the response phase, the probability that the opposition supports policy H declines over time due to the growth of direct cost. On the other hand, the probability *q* that the government selects policy H (that makes the opposition indifferent between supporting or criticizing the latter) remains unchanged, as the direct and indirect costs of the epidemic are unaffected by the behaviour of the opposition. As *p* becomes non-positive, the government is no more indifferent between playing H and M. Rather, it will play H with certainty (while the opposition will play M, i.e., it exploits the emergency to gain political advantage). However, the implementation of H will occur with a delay whose magnitude depends on the time needed by probability *p* to vanish during randomization.

This said, randomization will hardly represent a sound approach to political decision-making for outbreak control. For this case, game theory suggests, as an alternative ensuring a continued policy actions, to replace the mixed strategy by a compromise policy $\bar{HM}$ lying in between the two alternatives H and M. The leads to consider, for example, the average policy ${\bar{R}}_{HM}=qR_{H}+(1-q)R_{M}$. Therefore, if ${\bar{R}}_{HM}>1$, the adopted $\bar{HM}$ policy will remain mitigative, although stricter than the original M policy. This case therefore works as the one presented in the previous section: the resulting growth of direct costs will eventually yield to the emergence of H as a pure strategy Nash equilibrium. Clearly, the switch to H will occur with a larger delay compared with the original M policy (Figure 2, central row) and might yield a heavier legacy in terms of severe cases at the moment when the suppression policy H starts.

## 4 Discussion

This work attempts to expand the behavioural epidemiology of infectious diseases (2), by developing a conceptual framework for strategic political behaviours during the response to a threatening epidemic outbreak. Our motivations are mostly based on the COVID-19 experience. The primary motivation lies in the general assessment of global COVID-19 control as a failure on multiple levels, especially in Western countries (1). The second motivation lies in the COVID-19 tragedy in the Bergamo province, Northern Italy, during the first wave in February–March 2020 (13). Northern Italy was the first site worldwide experiencing a large-scale epidemic after China (12). Particularly in Bergamo, delays in the response yielded a catastrophe with +600% excess mortality (compared with previous years) in the province as a whole, but up to +2,000% in its most severely affected municipalities (13). Therefore, a further main motivation lies in the ERA (“early-rapid-aggressive”) principle (7), which should represent the basis of any response to a severe outbreak but—during COVID-19—failed to be applied in many sites (1, 11).

Which are the true failures underlying the Bergamo catastrophe is still unclear, despite an ongoing judiciary inquiry (21). However, anecdotal and scattered evidence is available on the pressure enacted by politicians and lobbies to avoid severe closures in the area, despite clear evidence of an ongoing major epidemic. This suggests that a strategic interaction has occurred while the response decision-making was ongoing (21–25).

Based on previous motivations, we developed a game-theoretic framework for unfolding the strategic determinants of public decision-making that potentially underlie the political response to an outbreak. The framework considered a government and its opposition, both trading off between political consensus and the protection of health, as well as a myopic economic lobby always prioritizing economic costs to health. The framework was used to investigate when the enaction of an ERA policy will be delayed or de-potentiated depending on the degrees of mutual responsibility of political actors and, more generally, how political behaviour interplays with epidemiological and policy making conditions during an outbreak.

Our results indicate that an ERA policy, i.e., a timely suppression policy, will always (never) be enacted after evidence of sustained transmission when the government is responsible (irresponsible), i.e., it always (never) puts the public good of health before consensus. In addition, ERA will also be enacted by a partly responsible government provided it is supported by a responsible opposition. Instead, ERA has no chance of being enacted by a partly responsible government when the opposition is irresponsible, i.e., only pursuing consensus. In this case, the fear of a loss of consensus due to the critics of the opposition will force the government to proceed with a mitigation policy only. However, epidemic growth will eventually dominate the government's payoff, so that transition to suppression will always occur. That is, the government (and the opposition) will unavoidably be forced by epidemic growth to switch towards a higher degree of “responsibility” towards citizens. Both the delay in the transition to suppression as well as the “legacy” due to the initial epoch of mild control will depend in a rich manner on the interplay between policy and epidemiological parameters (such as the time delay between infection and onset of severe disease) so that the possibly harmful effects of the delays in implementing a suppression policy might be amplified by the characteristics of infection.

Furthermore, a non-trivial situation of stall in the government's decision-making arises when both the government and the opposition are partly responsible. This case generates a discoordination game where both the government and the opposition have an *ex post* incentive to change their strategy after observing the choice of the rival. Epidemic costs are expected again to eventually force the escape from the stall but at the price of an increasing delay in the transition to suppression.

Whether such strategic interactions in the COVID-19 response decision-making were a major contributor to the intervention delay and to the ensuing catastrophic epidemic in Bergamo or not, would require further investigation over materials and data not easily accessible. Nonetheless, this work brings theoretical evidence and qualitative insights on plausible political mechanisms that might force delayed or mild responses to a threat, namely the strategic interaction of lobbies only prioritizing indirect costs, with decision makers initially more concerned with political consensus than health protection.

During the COVID-19 pandemic, an increasing number of works used game-theoretical approaches to model epidemic trends, the public response, and the role of behavioural changes; see (5, 40–47) and references therein. However, most such efforts maintained the traditional focus on uncoordinated behaviour of individuals. From this standpoint, this work represents a first effort to broaden the behavioural epidemiology of infectious diseases (2–5) by explicitly combining strategic political behaviour in decision-making with infection modelling.

Our approach clearly calls for a number of refinements. Although we already pinpointed above the possible data issues, empirical work would be important to better identify the roles of harmful political behaviour in undermining COVID-19 control, along the general suggestions in Sachs et al. (1). Relatedly, COVID-19 has seen the birth of new research going beyond traditional analysis of policy effectiveness and aiming to identify the true causal roles played by government-level measures vs. individual-level behavioural changes during the COVID-19 epidemic (48, 49). This approach could also be fruitful for empirically disentangling the effects of strategic political interactions as those analyzed, but only theoretically, in this work.

Also, combining this framework with spontaneous individual behaviour (2–5) would allow one to account for phenomena, as the adherence to public measures, which in polarized political systems can be dramatically oriented by political competition and governance (15).

Clearly, the realm of actual political decision-making of the response to COVID-19 was largely more complex, with many more actors, than proposed in this work. This was debated in an endless list of works which we can only sample, including (a) the way single charismatic political leaders used science in their communication (50); (b) the role of scientific committees in political decision-making (51) and the emerging conflict between relevance and usefulness of scientific advice, on the one hand, and expectation of political neutrality, on the other hand (52); (c) the role of pharmaceutical companies and the dramatic increase of their lobbying power during the COVID-19 pandemic, both in draining public resources in the pre-vaccination period as well as in the global distribution of vaccines (53). This lobbying power resulted a major determinant of the inequity in the global vaccine distribution (54), as also noted in Sachs et al. (1); (d) the role of supra-national institutions like the European Union in addressing socio-economic policy during major emergencies (55), particularly the massive effort to exit from the COVID-19 crisis (56); (e) the issue of poor international coordination as a main source of global control failure (1), including the role and mistakes of international institutions, as the WHO (1, 57, 58); (f) the tensions between central and local governments, also documented in the Bergamo crisis, that represented serious barriers in the efficacy of the overall response (1, 15, 59); (g) the role of imitation between governments facing different epidemic stages; for example, governments may imitate each other's policy to enforce the image of public health authority and contextually reduce the opportunities for opposition or conspiracy theories, etc.

Further, the proposed political game lacks true dynamics (beyond the exogenous evolution of costs), while the entire COVID-19 experience has been a continuous interplay of novelties on the epidemic side (e.g., the onset of new variants) feeding back on the decision-making system and the related issues of consensus under information asymmetries that represent key topics of game theory (27, 33).

This said, the multi-scale global failure in COVID-19 control and the lack of consensus that still surround the main response options (i.e., the elimination-suppression-mitigation trichotomy) witness the importance of considering the political layer in response and especially in preparedness activities. As for the latter, the present work can be also considered an exploration into the issues of top-level decision-making under emergency circumstances, and future pandemic plans should necessarily include agreed rules providing, beyond technical indications, ethical statements committing political parts to cooperation.

## Data Availability

The original contributions presented in the study are included in the article/Supplementary Material, further inquiries can be directed to the corresponding author.

## Footnotes

^1^The switch is not expected only for $\alpha_{G}=0$, yielding $B-C+(IC_{H}(t)-IC_{M}(t))>0$ as by definition, H always yields higher indirect costs than M. However, this contradicts the hypothesis of a partly responsible government.
