# Supplementary material for: “Early, rapid, aggressive”: when strategic interactions between governments, opposition, and lobbies can hinder effective responses to epidemics
Source: Front Epidemiol. 2025 Jun 18;5:1593883. doi: 10.3389/fepid.2025.1593883 (PMC12213477; doi:10.3389/fepid.2025.1593883)
Supplement: Supplementary file 1 [file Supplementaryfile1.docx]

Supplementary Material to:

**“Early, rapid, aggressive”: when strategic interactions between governments, oppositions and lobbies can delay effective responses to epidemics.**

This Supplementary material is two parts. The first part provides further details and intuitions on the game-theoretic representation of the political game reported in the main text. The second part aims to provide a broader perspective of the topic by deriving our political game from a general, fully micro-funded, economic framework of election competition in democratic systems.

***Part 1: further details on the political game.***

1. ***Characterization and simplification of the payoff matrix***

Here we prove the general statement (see section 2.2 of main text) by which – regardless of the agents’ degrees of responsibility – if the lobby’s best reaction to government strategy is to always play N, (i.e., the lobby is irresponsible), then the underlying (2x2x2) payoff matrix simplifies to the compact (2x2) representation adopted in the main text. To prove this, let us depart from the fully general structure of the payoff matrix during the pre-epidemic phase. This can be specified into the following (2x4) representation (Supplementary Table 1) contrasting the two possible strategies of the Government ($H,M$) with the four possible “combined” strategies of the opposition and the lobby, namely ($H,H$),($H,M$),($M,H$), $(M,M)$:

|  | **Opposition and Lobby** | | | |
| --- | --- | --- | --- | --- |
| **Government** | H,H | H,M | M,H | M,M |
| H | $P_{gov}^{pre}(H,H,H)$  $P_{opp}^{pre}(H,H,H)$  $P_{lobby}^{pre}(H,H,H)$ | $P_{gov}^{pre}(H,H,M)$  $P_{opp}^{pre}(H,H,M)$  $P_{lobby}^{pre}(H,H,M)$ | $P_{gov}^{pre}(H,M,H)$  $P_{opp}^{pre}(H,M,H)$  $P_{lobby}^{pre}(H,M,H)$ | $P_{gov}^{pre}(H,M,M)$  $P_{opp}^{pre}(H,M,M)$  $P_{lobby}^{pre}(H,M,M)$ |
| M | $P_{gov}^{pre}(M,H,H)$  $P_{opp}^{pre}(M,H,H)$  $P_{lobby}^{pre}(M,H,H)$ | $P_{gov}^{pre}(M,H,M)$  $P_{opp}^{pre}(M,H,M)$  $P_{lobby}^{pre}(M,H,M)$ | $P_{gov}^{pre}(M,M,H)$  $P_{opp}^{pre}(M,M,H)$  $P_{lobby}^{pre}(M,M,H)$ | $P_{gov}^{pre}(M,M,M)$  $P_{opp}^{pre}(M,M,M)$  $P_{lobby}^{pre}(M,M,M)$ |

*Supplementary Table 1. A fully general structure of the payoff matrix during the pre-intervention phase, including the possible strategies of the government (first column), against the possible combinations of strategies of the opposition and the lobby jointly considered.*

By the definition of irresponsible lobby (main text, section 2.1), the payoff of the lobby in (H,H,M) is higher than in (H,H,H). Similarly, (M,H,M) yields a higher payoff compared to (M,H,H). Finally, the outcomes (H,M,M) and (M,M,M) are respectively better than (H,M,H) and (M,M,H) from the lobby’s standpoint. In other words, columns 1 and 3 cannot form any Nash equilibrium, as the lobby would change its strategy towards the most profitable outcome of columns 2 and 4, respectively. Therefore, we can collapse the original eight configurations of the general problem (reported in *Supplementary Table* ***1***) into the compact (2x2) representation of Table 1 of the main text.

Furthermore, given that the lobby has a unique level of responsibility (i.e., it is always irresponsible), the number of possible political games resulting by combining the degree of responsibility of all agents, is simply the 3x3x1=9 games. These nine games correspond to all possible pairs of responsibility levels of the government and the opposition.

1. ***Main result of the pre-intervention political game***

The main result of the political game during the pre-intervention phase, that was phrased in the Results (section 3.1 of the main text), is reported in the Table below for each of the possible degrees of responsibility of the government and the opposition. Irrespective of the level of responsibility of the oppositions, all columns of the first (last) line obviously yield to the implementation of policy H (policy M), as the latter is the dominant strategy for the responsible (irresponsible) government (see the main text).

| ***Opposition***  ***Government*** | ***Responsible*** | ***Partly responsible*** | ***Irresponsible*** |
| --- | --- | --- | --- |
| ***Responsible*** | *Yes* | *Yes* | *Yes* |
| ***Partly responsible*** | *Yes* | *Yes/No* | *No* |
| ***Irresponsible*** | *No* | *No* | *No* |

*Supplementary Table 2. The pre-intervention phase. Outcomes of the strategic interaction between government and opposition in terms of their mutual degrees of responsibility. “Yes” identifies the propensity of the government to to immediately start policy H upon evidence of sustained transmission*

1. ***The dis-coordination game: proof of the mixed strategy equilibrium and the pivotal role of the lobby***

Here, we demonstrate and provide a number of details on the case of the dis-coordination game arising when both the government and the opposition are quite responsible. In equilibrium, the probability p that the opposition will support the ERA policy shall be such that the government is indifferent between adopting it or choosing instead the mild one. Formally, this implies equalizing the government expected payoff from playing H (resulting in (H,H) with probability $p$ and (H,M) with complementary probability) and that from playing M (resulting in (M,H) and (M,M) with probabilities $p$ and $1-p$, respectively)

$$Ap+C\left( 1-p \right)=Dp+\left( 1-p \right)B$$

Solving for p:

$$p=\frac{B-C}{(A+B-C-D)}$$

Likewise, the probability q that the government will implement the ERA policy shall be such that the opposition is indifferent between supporting it or not:

$$Cq+\left( 1-q \right)B=Aq+\left( 1-q \right)D$$

Solving for q:

$$q=\frac{B-D}{\left( A+B-C-D \right)}$$

Notably, randomization means that the outcome of the single-shot of the game can be any of the four possibilities listed in Table 6 of the main text, ranging from the “desirable” (H,H), resulting in a timely intervention without political speculation with probability qp; (ii) a second best (H,M), having probability q(1-p) with the government implementing H but the opposition criticizing to obtain political advantage; (iii) the “bad” (M,H) (with probability (1-q)p) which is best for the lobby, quite good for the opposition which gains consensus but harmful for the government (and citizens’ health) which suffers both a poor outbreak control and a consensus loss; (iv) the “bad” (M,M) (with probability (1-q)(1-p)) in which both the government and the opposition are captured by the lobby (the true winner of the game), and the government is able to avoid a political loss thanks to the bad strategy adopted by the opposition, which is the loser of the game together with the citizens.

Notably, the harmful outcome (M,M) is possible, despite no fully irresponsible players, because the lobby, though lacking strategic relevance, is pivotal in the consensus process. This causes a misalignment between the objective of the government, which would like to achieve a consensus with the opposition in order to intervene without popularity loss, and that of the opposition, which would like that the government implements policy H to mitigate the outbreak having the possibility criticise it to gain electoral support.

1. ***Removing simultaneity: a Stackelberg approach***

All games considered in the manuscript are simultaneous. Clearly, we expect that during emergency situations it will be the government that moves first, therefore playing as a Stackelberg leader in a Stackelberg game (Mas Colell, 1995), and other players then respond. It is easy to show that most our results are unaffected. Specifically, all dominance-solvable games (i.e., games where at least one player has a dominant strategy) yield the same outcome irrespective of the timing of the game. This is true for all cases but the quite responsible government vs quite responsible opposition. In this last case, backward induction shows that (H,M) is the only Sub-game Perfect Nash Equilibrium.

1. ***Further instances documenting the non-trivial role of the lobby***

There are many instances allowing to appreciate that the lack of “full” political responsibility does not imply that the lobby does not play any role in influencing the decision of the other players. For example, in a repeated game setting forward induction may lead the lobby to encourage the policy H if its support is critical in changing the policy implemented by the government in the next shot.

**References**

Mas-Colell, A., Whinston, M. D., & Green, J. R. (1995), *Microeconomic theory* (Vol. 1). New York: Oxford university press.

***Part 2: A micro-funded framework for the political game of epidemic response***

In this section we provide a broader perspective of our approach by showing that our simple political game of outbreak control, with three levels of responsibility namely, “responsible”, “partly responsible”, “irresponsible”, can actually be derived as a “stylised” outcome of a more general, fully (micro-)funded political framework where both consensus and citizens (i.e., voters) are explicitly considered. This general approach defines the actions of the different players over a continuous range of strategies and starts from first economic principles namely, (1) the utility functions of voters in a democratic society with free elections, (2) the preferences of political actors namely parties and lobbies, as well as (3) rational (that is, optimising) behaviour of all players. The approach presented here adapts recently published work (Carrozzo Magli et al 2025) which generalizes nowadays classical economic-political studies of electoral competitions i.e., the three voters’ probabilistic model by Acemoglu et al (2013) and De Feo and De Luca (2017) by adding heterogeneity between electors.

***The game-theoretic setup: general ideas***

We consider a two-stage political game dealing with the response to an emergent outbreak of a severe infectious diseases in a population of $n$ voters. In the first stage, the incumbent government (G) and its rival opposition O (in the sequel we will often denote them as “the two parties”) simultaneously commit to a policy aimed to mitigate the outbreak. In the second stage, citizens can express their assessment of the actions proposed by the two political actors through their vote in a free election under proportional representation. Moreover, an economic lobby can enact efforts to bring additional votes to the preferred party, contingent on the proposed policy. In the standard approach adopted by models of electoral competition, the party who wins the electoral race forms the new government and enacts the corresponding policy. In our setup, where the epidemic emergency breaks out without manageable warning, we assume that the policy commitment made by the incumbent is (partially) executed in the pre-election phase, thereby generating consequences observable by voters. The opposition, by contrast, announces its alternative without implementing it. This timing structure allows voters to assess actual policy outcomes rather than mere proposals.^[[1]](#footnote-1)^

In what follows we synthetically describe the behaviour of the different political actors (government, opposition, lobby, electors) by their payoff net utility, or payoff functions. . For ease of exposition, in some case we will also speak of loss (or cost) functions, which just represent the underlying payoff multiplied by $(-1)$. Next, we briefly present the clues for deriving the solution of the game. Finally, we provide an interpretation of the results of this “continuous strategies” game and suggest how this can be simplified into the simple framework with three levels of responsibility proposed in the main text.

***Payoff functions***

*The lobby*

We consider a typical economic lobby suffering costs from any, however mild, social distancing response policy due to the effects of resulting restrictions on economic activity. The ensuing loss (or “cost”) function is defined as follows:

| $\pi=-H^{G}P^{G}-H^{O}(1-P^{G})-\frac{1}{2w}V_{L}^{2},$ | (1) |
| --- | --- |

In (1), $H^{G}$ and $H^{O}$ represent the intensity of the intervention policy proposed by the (incumbent) government and the opposition, respectively, $P^{G}$ is the lobby’s perceived probability that the government is re-elected (${P^{O}+P}^{G}=1$) and $V_{L}$ is the number of votes moved by the lobby to, say, the opposition i.e., its effort in the political campaign, while $w$ is a measure of its lobbying power. The meaning of (1) is that the lobby suffers a cost whatever might be the policy action concretely proposed by the government or the opposition, which are weighted by the corresponding probability of re-election of either parties. Moreover, the lobby has a cost for moving votes from one party to the other one. The overall magnitude of this cost is inversely proportional to its lobbying power. Finally, policy “intensities” $H^{G}$ and $H^{O}$ are assumed to be “continuous” i.e., they take values in some appropriate interval ranging from a minimum to a maximum.

*Citizens*

Citizens (i.e., voters) are characterized by the following loss function:

| $U_{i}^{j}=-\frac{p_{t}L}{H^{j}}-cH^{j}+\varepsilon_{i}^{j} i=1,\ldots,n; j=G,O ,$ | (2) |
| --- | --- |

representing the loss that the generic voter (i.e., voter i) would suffer if the response is enacted by political party j.

In expression (2), $p_{t}$ represents the perceived probability of infection at time t (before policy intervention) $L$ is the average health cost of being infected including also the cost of severe disease, $cH^{j}$ represents the individual-level perceived cost of intervention (economic/social/psychological etc) and $\varepsilon_{i}^{j}$ is the so called “ideological proximity” (or affinity) of voter $i$ to party j. As the qualitative results of the present analysis are unaffected for $c=0$, since now on we will make this assumption. Note, in particular, that the individually perceived direct cost of the epidemic is directly proportional to the product $p_{t}L$ representing- in a citizen’s perspective - the expected cost of severe disease in the absence of intervention, and inversely proportional to the intensity of the policy proposed by either parties.

In line with the cited models of electoral competition, we assume that there are three types of electors whose groups are, for sake of simplicity, assumed to have equal size ($n$/3). The first type includes “partly ideologic” electors of party $G$ . They are characterized by $\varepsilon_{i}^{O}=\infty$ and $\varepsilon_{i}^{G}=0$ i.e., they will never vote for the opposition regardless of the actually proposed policy. Rather, they will vote for the incumbent government $G$ if the latter proposes a response policy that ensures them a minimum level $-T_{G}$ of utility in terms of health protection, and abstain otherwise. Such threshold level is assumed to take a uniform distribution over an appropriate interval $\left[ -\frac{1}{\alpha},0 \right]$. Similarly, partly ideologic electors of party O are characterized by $\varepsilon_{i}^{G}=\infty$, $\varepsilon_{i}^{O}=0$ and $-T_{O}\sim\left[ -\frac{1}{\beta},0 \right]$. The previous ones are typical technical hypotheses of electoral models (Acemoglu et al 2013) but their substantive meaning is simple: $\alpha$ and $\beta$ represent the voters’sensitivity to the topic of health protection from the consequences of the outbreak and therefore their responsiveness in the moment of election. In plain words, the larger $\alpha$ ($\beta)$, the more voters demand for severe mitigation policies Finally, the last type includes fully ideologic electors i.e., citizens that will always vote for their preferred party irrespective of the proposed policy (De Feo and De Luca 2017). This group is characterized $N^{G}$ and $N^{O}$ electors respectively voting for the government and the opposition. Without loss of generality, we assume that the difference $N^{G}-N^{O}$ is uniformly distributed over an appropriate interval $\left[ -\frac{1}{2\phi},\frac{1}{2\phi} \right]$, where $\phi$ measures the level of electoral competition. The implication of this hypothesis is simply understood: by taking large values of $\phi$, the range of the distribution of the difference $N^{G}-N^{O}$ is narrow, meaning that the (random) realizations $N^{G}-N^{O}$, which can either be negative or positive, will be small, implying that the competition is strong (or strongly uncertain).

Remarkably, the model could be easily extended to encompass also more pragmatic electors, who might vote for either parties depending on both ideological and policy considerations.

Overall, the expected numbers of votes for party G from group G (which is the sum of its “ideological” plus “pragmatic” components):

| $V_{G}=\frac{n}{3}\Pr\left( -\frac{p_{t}L}{H^{G}}>-T_{G} \right)=\frac{n}{3}\alpha\left( 1-\frac{p_{t}L}{H^{G}} \right)$ | (3) |
| --- | --- |

Similarly, the expected numbers of votes for party O from group O is:

| $V_{O}=\frac{n}{3}\Pr\left( -\frac{p_{t}L}{H^{O}}>-T_{O} \right)=\frac{n}{3}\beta\left( 1-\frac{p_{t}L}{H^{O}} \right)$ | (4) |
| --- | --- |

Note that the quantity $1-\frac{p_{t}L}{H^{G}}$ is always meaningful i.e., non-negative, due to previous hypotheses.

*The government and the opposition*

The objective functions of the two main political actors namely, the government and the opposition, are exactly represented by their probabilities $P^{G}$, $P^{O}$ (see equation (1)) of winning the next election round, which, being rational political actors, they want to maximise. Based on previous developments^[[2]](#footnote-2)^ , we can write these quantities as follows:

| $P^{G}=1-\phi\left[ \frac{n}{3}\beta\left( 1-\frac{p_{t}L}{H^{O}} \right)-\frac{n}{3}\alpha\left( 1-\frac{p_{t}L}{H^{G}} \right)+V_{L} \right]$ | (5) |
| --- | --- |
| $P^{O}=1-P^{G}=\phi\left[ \frac{n}{3}\beta\left( 1-\frac{p_{t}L}{H^{O}} \right)-\frac{n}{3}\alpha\left( 1-\frac{p_{t}L}{H^{G}} \right)+V_{L} \right]$ | (6) |

Relationships (5)-(6) allow to reformulate the lobby’s loss function (1) as:

| $\pi^{j}=(H^{G}-H^{O})\left\{ 1-\phi\left[ \frac{n}{3}\beta\left( 1-\frac{p_{t}L}{H^{O}} \right)-\frac{n}{3}\alpha\left( 1-\frac{p_{t}L}{H^{G}} \right)+V_{L} \right] \right\}-H^{G}-\frac{1}{2w}V_{L}^{2}$ | (7) |
| --- | --- |

The previous cost functions define a “game” because the net utility resulting from the strategies of each player (political parties, lobbies, citizens) depend also on the strategies adopted by all other players. We solve the game by backward induction. In other words, the solution is determined throughout two sequential stages. We first compute the lobby’s reaction function (i.e., the number of votes to deliver to the oppositions) to the policy proposed by the two parties. Second, contingent on the lobby’s reaction (as well as on the voters’ behaviour, as already determined in equations (3) and (4)) and accounting for the behaviour of the rival political competitor, we compute the optimal policy proposed by each party.

***Game solution: stage two***

As previously stated, we first determine the lobby’s reaction function. Minimizing the loss function of the lobby (equation (1)) with respect to $V_{L}$ taking as given the strategies of the other players, yields the following first order condition for having a minimum loss:

| $\hat{V}_{L}=\left( H^{G}-H^{O} \right)\phi w$ | (8) |
| --- | --- |

Plugging the latter expression into the payoff functions of the two political actors’, one gets:

| $P^{G}=1-\phi\left[ \frac{n}{3}\beta\left( 1-\frac{p_{t}L}{H^{O}} \right)-\frac{n}{3}\alpha\left( 1-\frac{p_{t}L}{H^{G}} \right)+\left( H^{G}-H^{O} \right)\phi w \right]$ | (9) |
| --- | --- |
| $P^{O}=\phi\left[ \frac{n}{3}\beta\left( 1-\frac{p_{t}L}{H^{O}} \right)-\frac{n}{3}\alpha\left( 1-\frac{p_{t}L}{H^{G}} \right)+\left( H^{G}-H^{O} \right)\phi w \right]$ | (10) |

These expressions represent the objective function of the two political parties conditional on the lobby’s best choice (8).

***Game solution: stage one***

Simultaneously maximizing expressions (9)-(10) one with respect to $H^{G}$ and $H^{O}$ and subsequently plugging the results back into the lobby’s best response (equation (8)), yields the game solution as the following set of first order conditions:

| ${H^{G}}^{*}=\sqrt{\frac{n}{3}\alpha\frac{p_{t}L}{\phi w}}$ | (11) |
| --- | --- |
| ${H^{O}}^{*}=\sqrt{\frac{n}{3}\beta\frac{p_{t}L}{\phi w}}$ | (12) |
| $V_{L}^{*}=\sqrt{\frac{np_{t}L\phi w(\alpha-\beta)}{3}}$ | (13) |

The equations (11)-(13), which are surprisingly simple given the richness of the framework and of the underlying hypotheses, provide the desired optimal intensities of the intervention policies against the outbreak – i.e., the intensities optimising the payoffs of the two parties, as well as the magnitude of the number of votes that the lobby is willing to move from one party to the other based on the actual policy choices. These optimal policies are expressed in terms of a number of parameters reflecting the political game but also the individual-level direct and indirect costs of the epidemic.

From these continuous-strategies equations, we note that, the lobby will increase its political effort against severe restrictions (i.e., in terms of the number of votes moved, equation (13)) the higher the direct cost expected from the epidemic ($p_{t}L$). Moreover, its political involvement grows in its lobbying power ($w$) as well as in the level of electoral competition ($\phi$) and in the difference between the policies proposed by the two parties.

As for political parties, they will propose stricter social distancing restrictions (equations (12) and (13)) the higher is the expected direct epidemic costs ($p_{t}L$) and the higher the concern ($\alpha or \beta$, respectively) of their ideological voters towards health issues related to the epidemic. However, the intensity of the proposed response is strictly decreasing in the level of electoral competition $\phi$ which reflects the uncertainty in the outcome of the incoming election and in the lobby’s power ($w$). In other words, uncertainty in the electoral competition and the power of the lobby are both factors that can reduce political consensus and therefore deteriorate the intensity of the response to the epidemic outbreak.

***From continuous to discrete strategies: the three levels of responsibility***

The formulation presented in the main text, which considered three levels of political responsibility (high, intermediate, low) can be obtained by a straightforward discretization of the continuous-strategy equilibrium characterized here by equations (11) and (12). In the continuous model (11) -(12), the equilibrium level of the intensity of the response to the epidemic that would be desired by either parties, depends on the relative importance played by considerations related to health, captured in the numerator of equations (11) and (12) (which include the individuals’ expected cost of the epidemic as well as the voters’ sensitivity to this topic) with respect to consensus considerations, which captured in the corresponding denominator. Thus, the position of both political parties on the “responsibility spectrum” can be interpreted as the result of this balance: high responsibility corresponds to the situation where public health concerns dominate electoral/consensus concerns, and vice versa.

Therefore, in order to sort-out the simple approach with three responsibility levels, we can simply partition the intensity of continuous responses into three discrete types only, namely low priority to health (“irresponsible”), middle (“partly responsible”) and high (“responsible”). Will it be enough to stop the pandemics spread? Crucially, the answer depends upon political (and lobby-related) considerations, as in the discoordinative game presented in the main text.

In particular, if we define a minimum level of response intensity such that the proposed policy is classified as H (and M otherwise), then -depending on the responsibility levels of the government and oppositions, all four combinations of strategies that have been proposed in the main text for the government and the opposition namely, (H,H), (H,M), (M,H) and (M,M) emerge. Instead, analogous to the main text, the strategy of the lobby is unaffected by its lobbying power: being able to move the desired number of votes, the lobby will always support the party proposing lower restrictions i.e., its preferred strategy will always be M. This allows to preserve all the analyses presented in the main next, from Nash equilibria to the dis-coordination case.

**Final remarks on the proposed approach**

The previous analysis has primarily focused on the phase that we termed the “pre-intervention” phase in the main text. The extension to the “intervention” phase can also consider indirect epidemic costs in an explicit manner, which is straightforward (e.g., by re-incorporating the individually perceived cost of the proposed policy $cH^{j}$that, for sake of simplicity, was set to zero here).

**References**

Acemoglu D, Robinson JA, Santos RJ. The monopoly of violence: Evidence from Colombia. JEEA, 11(S1):5–44, 2013.

Carrozzo Magli A, Righetto G, Schiavone A. Votes for Assets: Mafia, Elections and Misallocation of Confiscated Properties. Available at SSRN: https://ssrn.com/abstract=5219444 or http://dx.doi.org/10.2139/ssrn.5219444.

De Feo G, De Luca GD. Mafia in the ballot box. AEJ: Economic Policy, 9(3):134–167, 2017.

1. Most of the proposed statements should be intended in a broad sense. Clearly, we expect that setting elections during the most intense phase of a serious outbreak might be impractical, but any mechanism for ascertaining political preferences (e.g., a population -wide representative poll conducted in real time, as characteristic of modern democracies) might serve the purpose. [↑](#footnote-ref-1)
2. To derive equation (5) ((6) is just complementary) we note that the probability that the government obtains more votes than the oppositions is $\Pr\left( V_{G}+N_{G}>N_{O}+V_{O}+V_{L} \right)=1-Pr(N_{G}-N_{O}<V_{O}-V_{G}+V_{L})$. [↑](#footnote-ref-2)
